# Postoperative cognitive dysfunction: a concept analysis

**DOI:** 10.1007/s40520-024-02779-7

**Published:** 2024-06-21

**Authors:** Hesam Aldin Varpaei, Kousha Farhadi, Mostafa Mohammadi, Alireza Khafaee pour khamseh, Tahereh Mokhtari

**Affiliations:** 1 College of Nursing, Michigan State University, East Lansing, MI USA; 2 School of Medicine, Tehran University of Medical Sciences, Tehran, Iran; 3 Department of Critical Care, Imam Khomeini Hospital Complex, Tehran University of Medical Sciences, Tehran, Iran; 4 Faculty of Medicine, Islamic Azad University Tehran Medical Sciences, Tehran, Iran; 5 Department of Gynecology, School of Medicine, Shariati Hospital, Tehran University of Medical Sciences, Tehran, Iran

**Keywords:** Post-operative cognitive dysfunction, Delirium, Anesthesia, Neurocognitive disorders

## Abstract

**Background:**

Post-operative cognitive dysfunction (POCD) is a concern for clinicians that often presents post-surgery where generalized anesthesia has been used. Its prevalence ranges from 36.6% in young adults to 42.4% in older individuals. Conceptual clarity for POCD is lacking in the currently body literature. Our two-fold purpose of this concept analysis was to (1) critically appraise the various definitions, while also providing the best definition, of POCD and (2) narratively synthesize the attributes, surrogate or related terms, antecedents (risk factors), and consequences of the concept.

**Method:**

The reporting of our review was guided by the PRISMA statement and the 6-step evolutionary approach to concept analysis developed by Rodgers. Three databases, including Medline, CINAHL, and Web of Science, were searched to retrieve relevant literature on the concept of POCD. Two independent reviewers conducted abstract and full-text screening, data extraction, and appraisal. The review process yielded a final set of 86 eligible articles.

**Result:**

POCD was defined with varying severities ranging from subtle-to-extensive cognitive changes (1) affecting single or multiple cognitive domains that manifest following major surgery (2), is transient and reversible, and (3) may last for several weeks to years. The consequences of POCD may include impaired quality of life, resulting from withdrawal from the labor force, increased patients’ dependencies, cognitive decline, an elevated risk of dementia, rising healthcare costs, and eventual mortality.

**Conclusion:**

This review resulted in a refined definition and comprehensive analysis of POCD that can be useful to both researchers and clinicians. Future research is needed to refine the operational definitions of POCD so that they better represent the defining attributes of the concept.

**Supplementary Information:**

The online version contains supplementary material available at 10.1007/s40520-024-02779-7.

## Background

Cognitive functioning is an evolving process encompassing human life and is a continuous endeavor of learning, maintaining short-and long-term memory, and electing to facilitate executive functioning [1]. The term “cognitive dysfunction” refers to difficulties with selective and sustained attention, learning and memory problems, deficits in visual and auditory processing, and processing speed [2]. Cognitive dysfunction can be caused by a direct neurological phenomenon, such as a brain injury, or by a non-direct phenomenon, such as surgery [3].

Post-operative cognitive dysfunction (POCD) is a specific cognitive impairment that involves functional impairment of the nervous system’s activities, such as selective attention, vigilance, perception, learning, memory, executive function, verbal and language abilities, emotion, visuospatial, and visuomotor abilities. POCD occurs in the absence of head trauma or other brain-related injuries [4, 5]. POCD is a serious public health concern, with prevalence rates ranging from 36.6% in young adults to 42.4% in the elderly [6]. It can occur following major invasive procedures such as cardiac [7], non-cardiac [6, 8], and carotid surgery that are lengthy and intensive [9]. POCD is diagnosed using standardized neuropsychological assessment tools that are available to assess cognitive function both pre- and post-operatively. The most clinically used screening tests for evaluating the overall cognitive functioning of patients are the Mini-Mental State Examination (MMSE; [10]) and the Montreal Cognitive Assessment (MoCA; [11]). The most commonly used clinically for assessing specific domains of cognition function rather than the overall assessment of cognitive function, are neuropsychological test batteries. These include the Trail-Making Test A (assessing visual attention, processing speed, and executive function), Trail-Making Test B (assessing cognitive flexibility, set-shifting, and executive functions), Digit Span Forward (assessing short-term auditory memory and attention), Digit Span Backward tests (assessing working memory and cognitive flexibility), Wechsler Memory Scale (assessing short-term memory, episodic memory and visuo-spatial working memory), and the Wechsler Adult Intelligence Scale (assessing vocabulary, comprehension, arithmetic and reasoning skills) [12–14]. While this information is valuable, due to POCD’s nature consisting of subjective and objective aspects, the assessment and diagnosis of POCD require approaches that extend beyond sole reliance on standardized test results, such as incorporating patients’ subjective reports alongside the outcomes of neuropsychological tests for a comprehensive evaluation.

Previous studies [6, 7, 15–17] have focused on the variations in the timing of symptom manifestation and their influence on the assessment and diagnosis of POCD. As a result, POCD has been categorized into early (acute) and late-onset POCD. These timing distinctions become essential as it recognized that several health-related outcomes are associated with POCD, such as delayed recovery, dependency on financial assistance [18], decreased quality of life [19], and an increased risk of death [20].

Despite the possibility of adverse long-term effects of POCD, the phenomenon has yet to be assigned a Diagnostic and Statistical Manual of mental disorders (DSM 5-TR) category [21, 22] even in the latest fifth edition; and conceptual clarity of POCD needs to be improved. For example, different terms have been used interchangeably to describe POCD, such as post-operative cognitive decline and post-operative cognitive impairment. Even the 1st, 2nd and 3rd International Study Group of Post-Operative Cognitive Dysfunction (ISPOCD), the major scientific and scholarly association focusing on this phenomenon, has not provided a clear definition of POCD [5, 23, 24]. To address the notable gaps in research while advancing the state of the science, our two-fold purpose of this study was to: (1) critically appraise, discuss, and challenge the various meanings of POCD thus providing a befitting definition for both clinical and research purposes; and (2) narratively synthesize the attributes, surrogate or related terms, antecedents including risk factors, and consequences of the concept. Such analysis can potentially lead to the development of a conceptual framework that is testable, and to research that can promote improved patient care and enhanced clinical outcomes.

## Methods

### Design and data sources

Our concept analysis follows Rodgers’ evolutionary method [25], recommended for analyzing concepts that change over time with increasing research and those that vary across contextual circumstances. The chosen concept for analysis is POCD, for which surrogate terms were identified. The method comprises six steps: (1) identifying the concept of interest (2), selecting the appropriate context and sample (3), identifying antecedents, attributes, and consequences (4), evaluating data about the concept’s characteristics (5), providing an illustrative example, and (6) recognizing implications for its ongoing development. Studies were retrieved through searches in PubMed, Cumulative Index of Nursing and Allied Health Literature (CINAHL), and Web of Science databases, assisted by a master’s-prepared university-affiliated librarian. The keywords used for finding relevant articles were: “post-operative cognitive dysfunction,” “post-operative cognitive decline,” “POCD,” and “post-operative cognitive impairment.” Database searches were limited to publications after 1998, which was the year of the first ISPOCD convention.

**Fig. 1:**
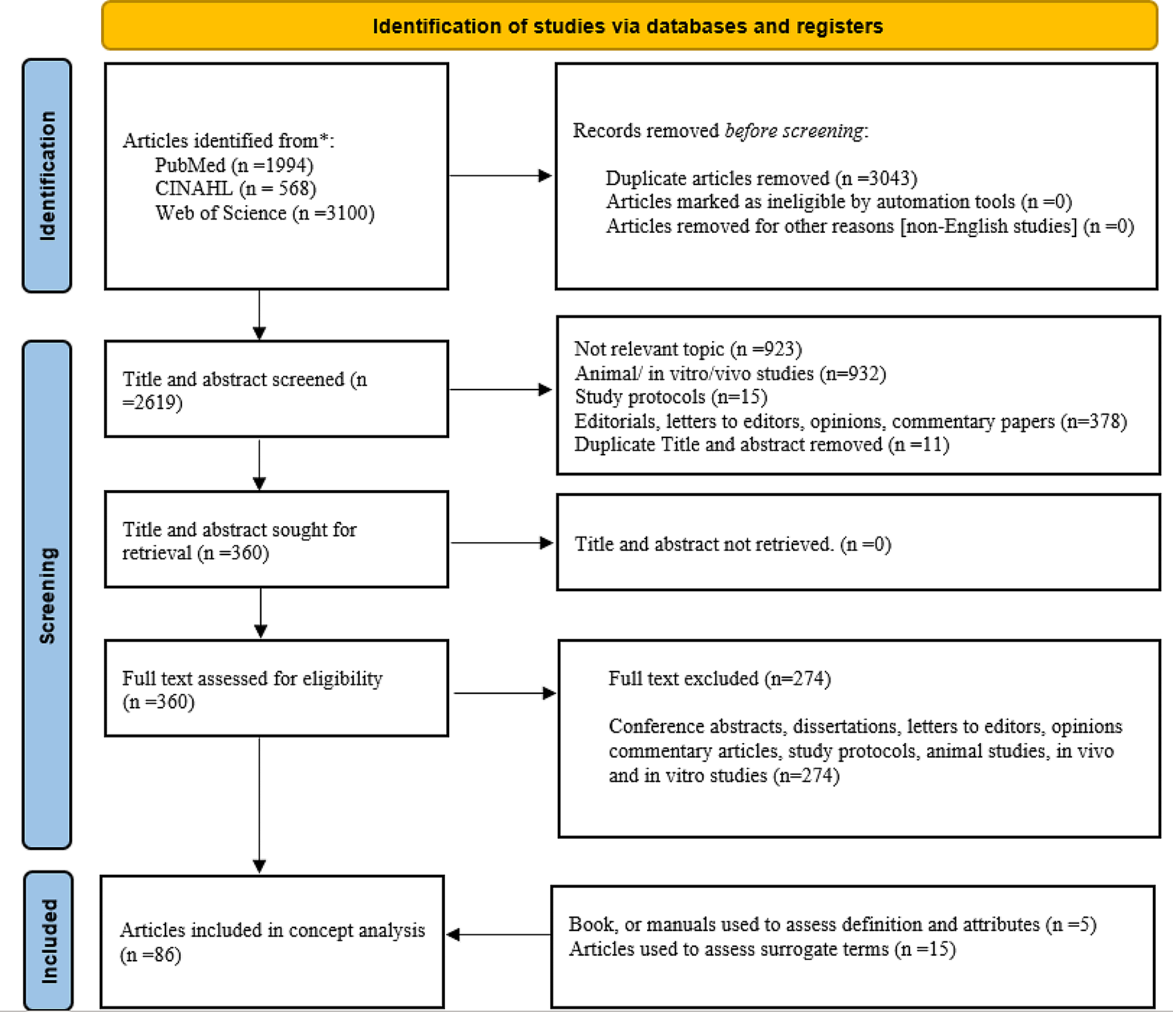
– PRISMA flow diagram

Inclusion criteria encompassed English-language quantitative original and review articles focusing on human subjects that used standard cognitive function assessment for POCD (using MoCA, MMSE, battery of tests, etc.), and provided definitions, assessed risk factors, or evaluated consequences (short and long terms) of POCD. Also, Webster’s dictionary, lezak’s neuropsychological assessment textbook, and DSM 5-TR were used to provide more accurate definitions of POCD and its attributes. Exclusion criteria encompassed conference abstracts, dissertations, letters to editors, opinions, commentary papers, and study protocols. Additionally, animal, in vivo and in vitro laboratory studies were excluded. The initial search yielded a total of 5.662 titles and abstracts from three sources of Web of Science, CINAHL, and PubMed. After exclusion for various reasons detailed in Figs. 1 and 86 studies were eligible for inclusion in the concept analysis (Fig. 1).

Two independent reviewers (HV and TM) screened all titles, abstracts, and full-text articles. In the case of any discrepancies, the two reviewers discussed the different views on certain points and came to an agreement. The goal was to include an article that defined POCD (attributes) and/or identified risk factors (antecedents) and/or consequences. Lastly, a conceptual and operational definition was formulated. HV and AKPK entered all relevant information related to each area into an Excel 2020 database and discussed the findings with each other. HV and AKPK discussed all findings to ensure consistency and clarity. Four related (surrogate) terms were found in the literature, and they searched separately to find the definition and distinction by the first author.

## Results

Of the 84 articles included in this concept analysis (see appendix 1), 33.3% were from the USA (*n* = 28), 19% from China (*n* = 16), 8.3% from Denmark (*n* = 7), 7.1% from Germany (*n* = 6), and 4.7% from the UK (*n* = 4). The remaining 27.6% represented various countries (*n* = 23). In terms of study designs, 52.4% were reviews (*n* = 44), 38% were cohort studies (*n* = 32), 7.1% were randomized controlled trials (RCT; *n* = 6), and 2.5% used other designs (*n* = 2).

### Cognitive function and dysfunction

The DSM 5-TR identifies complex attention, executive function, learning and memory, language, perceptual-motor control, and social cognition as the six essential domains of cognitive function [22].

Attention is the most fundamental element of effective cognitive function [22, 26], and may be categorized into sustained, selective, and sustained selective types. Sustained attention is the ability to maintain sensitivity to stimuli, while selective attention is the ability to process a certain part of sensory input while omitting others. Sustained selective attention is the ability to process certain stimuli at the expense of others in a span of time, which some believe is a framework for executive function development [27]. Executive function refers to the capacity to complete complicated tasks such as planning, reasoning, problem solving, and adaptation to complex situations. It also includes working memory, inhibitory control, and cognitive flexibility. Executive function also controls other cognitive domains such as attention and memory [28]. Memory and learning are mutually interdependent and can be categorized by recent (short-term), rapid, recall, recognition, and long-term memory. Language or semantic memory is reflective of verbal fluency, grammar use, and syntax. Other important aspects of cognition include perceptual-motor control, which refers to the ability to understand visual perceptions. Finally, social cognition reflects emotion recognition and the ability to discern other’s mental status and feeling states. Cognitive processing is viewed in the cognitive psychology field as a series of sequential phases during which sensory data are converted, reduced or simplified, elaborated, stored, retrieved, and used for effective function and survival [29, 30]. According to DSM 5-TR, Neuropsychological testing typically shows cognitive deficits in areas such as executive function, verbal memory, and speed of processing.

In the current literature, three words are primarily used to describe cognitive changes: dysfunction, decline, and impairment. In Webster’s dictionary [30], *dysfunction* is defined as damaged (or impaired) unhealthy functioning (abnormal) that can be seen in interpersonal interactions or group interactions. Because POCD is often referred to with other deficit terms, such as decline and impairment, these terms are viewed as being related. Per Webster’s dictionary, the word *decline* (noun) refers to a “gradual physical or mental sinking and wasting away, or the period during which something is deteriorating or approaching its end (downward slope)”. Further, the word *impairment* is defined as “diminishment or loss of function or ability” [30]. Thus, the words “dysfunction” and “impairment” are considered approximate, except for some minor differences. Cognitive dysfunction typically describes temporary or reversible alterations in cognitive function, often stemming from factors like fatigue, medication side effects, or acute illnesses, and generally improves when the underlying cause is addressed. Cognitive impairment, on the other hand, suggests more enduring and noticeable deficits in cognitive abilities, often linked to conditions such as mild neurocognitive disorder (NCD), where cognitive issues exceed normal age-related changes but do not meet the criteria for dementia. In contrast, cognitive decline signifies a gradual and progressive deterioration in cognitive function over time, often associated with age-related changes but also seen in conditions like Alzheimer’s disease and various dementias. Cognitive decline tends to be an ongoing process and can ultimately become pervasive. These distinctions help clarify the nature and potential reversibility of cognitive changes.

In addressing the subject at hand, numerous studies have reviewed POCD definitions. Newman et al., in their review, defined POCD as a’significant’ change in postoperative cognition compared to the preoperative period, a definition more consistent than POD [4]. Liu et al. [13] portrayed POCD as a reversible form of MCI, while Needham et al. [12] described POCD as a mild neurocognitive disorder developing between 7 days and 1 year from surgery. Other studies have suggested that POCD can last up to 5 years, and even 7.5 years postoperatively. However, a study conducted by Steinmetz et al. [31] on 686 patients undergoing non-cardiac surgery indicated that POCD is largely reversible over time [12, 13, 24, 31, 32].

This uncertainty in the definition of POCD is manifested in the methods used by researchers to evaluate the incidence of POCD. For instance, Steinmetz et al. utilized the Visual Verbal Learning Test, the Concept Shifting Test, the Stroop Color Word Interference Test, and the Letter Digit Coding Test and calculated the change from the preoperative baseline while also comparing the normative results from healthy individuals to count for the learning effect [33]. Although Evered et al. [32] also benefited from the use of results obtained from healthy individuals, their neurocognitive battery measurement consisted of the Consortium to Establish a Registry for Alzheimer’s Disease-Auditory Verbal Learning Test, the Digit-Symbol Substitution Test, Trail Making Tests A and B, the Controlled Oral Word Association Test, the Semantic Fluency Test, the Grooved Pegboard Test, and the National Adult Reading Test, which is different from the Steinmetz study [31]. Newman et al. used a battery of five tests, comprising the Short Story module of the Randt memory test, the Digit Span and Digit Symbol subtests of the Wechsler Adult Intelligence Scale-Revised test, the Benton Revised Visual Retention test, and the Trail-Making test B [34].

### Defining attributes

As noted in Table 1, the defining attributes (consistent conceptual characteristics) of POCD in patients post-surgery are as follows: (1) manifestation following after the acute phase (4–6 weeks), surpassing the usual time needed for recovery from the effects of surgery, which differs from surgery to surgery [35, 36] of the post-operative period [12]; (2) subtle- [37–39] to-extensive cognitive change (continuum of negative impact); (3) affects single or multiple cognitive domains [4, 31, 33, 37]; (4) reversible nature [12, 38, 31, 41]; and (5) may last for several days to years [5, 23, 32, 34, 37, 41] Implicitly, POCD should be assessed and detected by neuropsychological tests [5, 12–14], such as MoCA, MMSE, Digit Span test [34], trail making test [32], and other standardized neuropsychological battery of tests.

**Table 1:** – Defining attributes (characteristics) of POCD

Manifestation following the acute phase of the post-operative period	- Onset after the acute period of surgery [12, 35, 36].
Subtle-to-extensive cognitive change (varying severity)	- Slower progression than post-operative delirium (POD) [37] Subtle nature [38,39].
Affects a single or multiple cognitive domains	- Short- and long-term memory and/or executive domains [4, 40]. - Affects single or multiple domains [33, 37].
Reversible nature	- POCD is usually self-restricted, recoverable [12], and reversible [31, 33].
May last for several days to years	- Several days [5, 23, 41] and up to 1–7 years [5, 34, 38, 42].
***Antecedents (***risk factors)	
*Risk factors*	*Descriptions and sources*
Lower educational level, illiteracy, and older age *(Non-modifiable)*	- Older patients [20, 42–44] are more likely to develop POCD than younger ones. - A low level of education [45, 46] or illiteracy [46, 47] is associated with POCD.
Alcohol use disorder *(Non-modifiable)*	- Alcohol dependency contributes to developing POCD [48, 49].
Genetic factors *(Non-modifiable)*	- Hereditary factors may predispose patients to POCD [47, 50].
Preexisting cognitive impairment *(Non-modifiable)*	- The presence of a cognitive disorder before surgery increases the risk of POCD [51, 52, 53].
Surgical types and techniques *(Modifiable)*	- POCD occurs after major surgeries such as open-heart surgery [53, 54], total hip replacement [55, 56], carotid surgery [57–59], and thoracic surgery [60, 61]. - In cardiac surgery, off-pump coronary artery bypass grafting (OPAC) is associated with a reduced risk of POCD and is linked to cognitive improvement after cardiac surgery [62]. - Nonetheless, some preliminary findings suggest that the incidence of POCD after on-pump coronary artery bypass graft (CABG) is lower than after off-pump CABG. Still, the difference is not statistically significant [63].
Intraoperative management of homeostasis parameters *(Modifiable)*	- Normothermia and keeping the mean arterial pressure > 70 mmHg during surgery is a preventative measure to prevent POCD in cardiac surgery [64]. - Considering a short cardiopulmonary bypass (CPB) time, maintaining a mean arterial pressure < 80 mmHg during surgery may be beneficial for older patients as a POCD preventative measure [65]. However, some studies suggest that maintaining a lower mean arterial pressure (MAP) during surgery has no significant impact on POCD [66, 67].
Type of anesthesia *(Modifiable)*	- Total intravenous anesthesia (TIVA), in comparison to regional anesthesia (RA), may increase the risk of POCD (particularly in geriatric populations), but it may not be a risk factor for POD [68]. No differences between RA and TIVA were noted in short-term cognitive dysfunction [69]. - Inhalational anesthesia, compared to TIVA, is associated with an increased risk of POCD [70]. - Propofol-based anesthesia reduces the odds of POCD [71, 72]. - Intraoperative dexmedetomidine reduces the risk of POCD [73–75]. - Intraoperative infusion of ketamine [76] and lidocaine [44] prevent POD and POCD. - Monitoring the depth of anesthesia bispectral index prevents POCD [77], but there is no link between the depth of anesthesia and POCD [78]. Electroencephalography and regional cerebral oxygen saturation monitoring are preventive measures to reduce the risk of POCD [79].
Comorbidities *(Modifiable)*	- Diabetes was reported to be a risk factor for developing POCD. It was reported that diabetic patients had a 1.84-fold increased risk of POCD [80, 81–83]. - Hypertension and a higher BMI (obesity) were not significant risk factors [81, 84]. - Higher blood glucose (hyperglycemia) during surgery is also suggested to be a predictor of POCD [85, 86] so hyperglycemia, especially in non-diabetic patients, can be a risk factor. However, a large-scale RCT the noted the use of intraoperative insulin does not reduce neurobehavioral deficits [86], and found that aggressive control of hyperglycemia will not improve neurocognitive outcome. - Preoperative insulin resistance is a strong predictor of the development of POCD [87].
Post-operative delirium (POD) *(Modifiable)*	- POD can significantly increase the risk of POCD [6, 41, 88–90].
***Summary of consequences (outcomes)***	
*Characteristics*	*Describing and source*
Impaired quality of life	- Withdrawal from the labor force [18] and increasing patient dependency on doing activities of daily living [6, 18] and, as a result, impairment in quality of life [8, 91, 92]. - Low quality of life is linked to cognitive impairment following heart surgery [24, 34, 93].
Cognitive decline	- The defect in cognitive function sometimes tends to be chronic and become permanent, which refers to cognitive decline [32, 34, 94–97].
Increased risk of dementia	- Developing POCD is associated with an increased risk of dementia in elderly populations [31, 97].
Increasing healthcare costs	- Recently, it was found that developing POCD may increase healthcare costs [98, 99].
Eventual mortality	- POCD is associated with an increased risk of overall mortality [6, 33, 98–101].

### Related concepts/surrogate terms

#### Post-operative delirium (POD)

POD is an acute state of fluctuating and altered consciousness due to underlying internal and external factors such as anesthetics, pain, and cerebral hypoperfusion [102]. A critical element in managing POD involves appropriately identifying and addressing the condition’s underlying cause(s). Delirium is defined as “a disruption in attention (i.e., diminished capacity to direct, concentrate, sustain, and change in focus of attention) and awareness (reduced orientation to the environment)” or “additional disturbances in cognition,” as noted in the DSM 5-TR [22] manual. The incidence of POD was reported to range from 5 to 40% [22, 103, 104].

#### Major neurocognitive disorder (dementia)

Formerly known as dementia, major NCD is any progressive and irreversible cognitive disorder characterized by a cognitive deterioration that interrupts a person’s ability to perform various occupational, home, or social tasks. Dementia is seen as pathological changes in the brain with several potential causes, as opposed to a distinct illness or syndrome [105, 106]. Dementia occurs as part of a spectrum of diseases that include Alzheimer’s, vascular (occurs with stroke and diabetes) dementia, dementia with Lewy bodies (DWLB), Parkinson’s, and mixed varieties [107].

#### Mild neurocognitive disorder

Formerly known as mild cognitive impairment, mild NCD refers to the period between age-related normal cognition changes and the onset of dementia symptoms [108]. Mild NCD is a cognitive impairment with little interference in everyday instrumental tasks, thus different from dementia [109, 110]. Mild NCD can be treated or prevented from progressing to a more severe stage [111], which can be considered a reversible condition. Mild NCD is not associated with surgery per se but can occur as a result of aging (degenerative diseases) and changes in the environment (such as moving to a nursing home) [112].

Diagnosing mild neurocognitive disorder (NCD) is applicable when composite scores in multiple domains deviate 1–2 standard deviation from normal, and major NCD is when the score is 2 or more standard deviation from the mean [22].

#### Cognitive vitality (CV)

CV refers to an individual’s capacity to adjust to cognitive changes and maintain an optimal level of cognitive functioning, life satisfaction, and independent living by effectively balancing their energy and personal resources. CV serves as a compensatory mechanism to adapt against the cognitive decline of aging. Although CV isn’t measured in a singular manner, its scope can be outlined by five essential domains: (1) physiological and metabolic health; (2) physical capability; (3) cognitive function; (4) psychological well-being; and (5) social well-being [113, 102]. CV is closely related to resilience and may reflect an individual’s capacity to recover from assaults such as surgery; it can increase the quality of life and result in healthy longevity [114, 115].

### Antecedents (risk factors)

The risk factors of POCD, as mentioned in Table 1, can be separated in 3 groups. First, lower educational level [45, 46], illiteracy [46, 47], and older age [20, 42–44] that are in relation to lower cognitive reserve ; Second is operation-related risk factors such as, surgical types and techniques (like cardiopulmonary bypass) [43, 44, 46, 53, 54–63], intraoperative management of homeostasis parameters (cerebral oxygenation, body temperature, blood glucose, and blood pressure) [64–67, 76, 77, 79] and type of anesthesia [48, 65–79, 105]. The third group consists of other perioperative risk factors such as alcohol use disorder [48, 49], genetic factors [46, 47, 50], preexisting cognitive impairment [51, 116], comorbidities [80, 81, 83–87, 117], and POD [41, 88–90].

### Consequences (outcomes)

Outcomes of POCD may include impaired quality of life [8, 24, 34, 91–93], which may result from withdrawal from the labor force with increased patients’ dependencies [6, 18]; cognitive decline [32, 34, 93–97]; increased risk of dementia [31, 97]; increasing healthcare costs [98, 99]; and eventual mortality [6, 18, 96, 98–101].

#### Conceptual definition of POCD

**Fig. 2:**
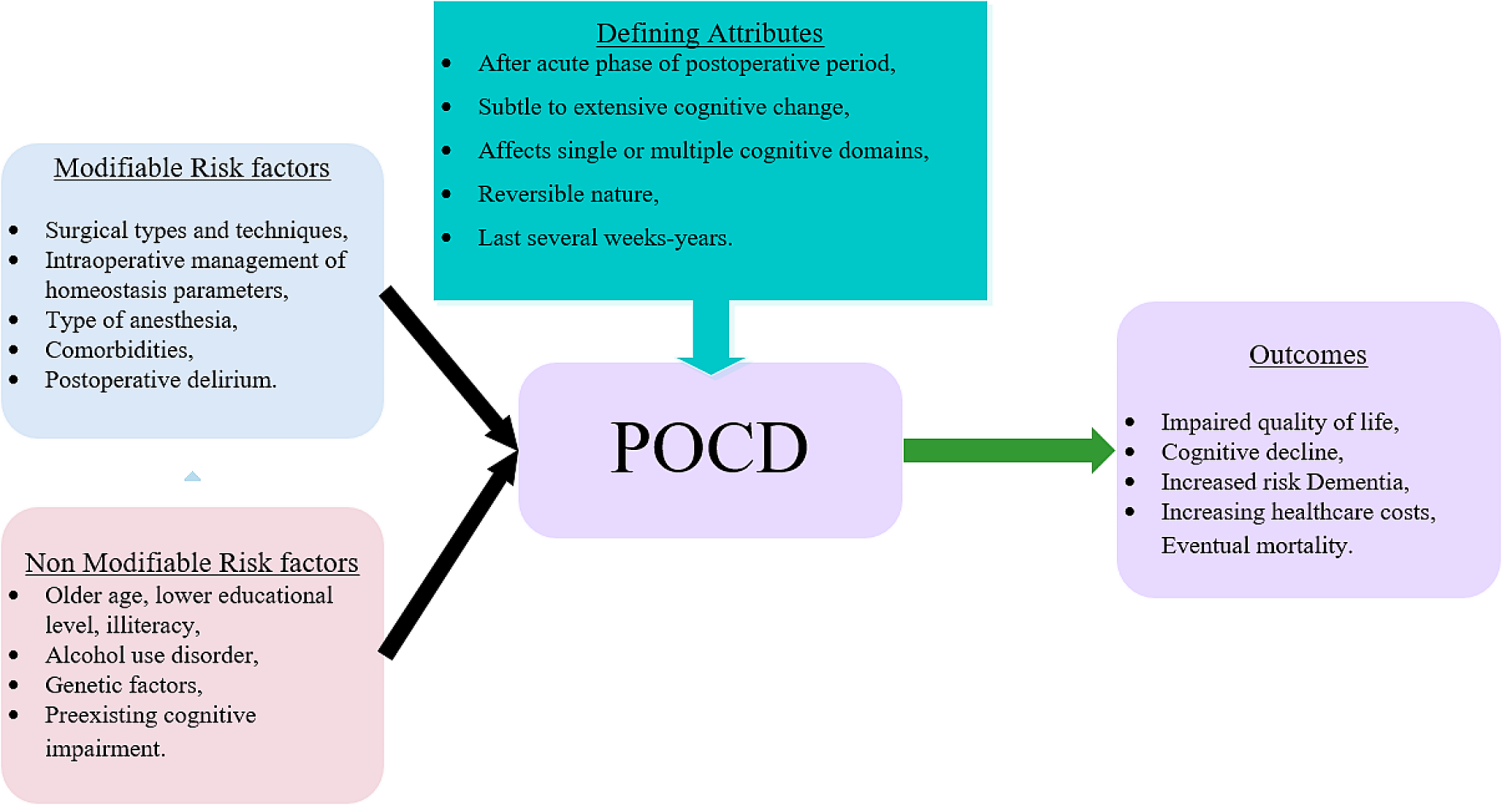
**–** POCD risk factors, defining attributes and outcomes diagram

Figure 2 provides a comprehensive definition of POCD, encompassing modifiable factors (those that can be manipulated by healthcare providers, i.e., surgeon, anesthesiologist, or nurse practitioner, to reduce the risk of incidence) and nonmodifiable factors (those that can’t be changed prior to or during surgery). The definition includes risk factors, defining attributes, and outcomes established based on previously discussed research, offering a thorough insight into POCD.

POCD manifests following the acute phase (4–6 weeks), surpassing the usual time needed for recovery from the effects of surgery, which differs from surgery to surgery. The post-operative period involves a subtle-to-extensive cognitive change (continuum of negative impact) that affects single or multiple cognitive domains, is reversible, and may last for several days to years. Post-Operative Delirium (POD), distinct from POCD, involves acute confusion and disorientation following surgery, requiring immediate medical attention. Since POD primarily entails dysfunction in attention and awareness, while POCD involves dysfunction in memory and executive function with or without other cognitive function domains, it is suggested to separate POD and POCD. Mild neurocognitive disorder, previously known as mild cognitive impairment, signifies cognitive difficulties that exceed normal age-related changes but fall short of dementia, typically involving memory problems. In contrast, major neurocognitive disorder, commonly referred to as dementia, is a severe and often irreversible condition characterized by significant cognitive decline that impedes daily functioning. This decline can stem from various underlying causes like Alzheimer’s disease and vascular dementia. These terms collectively reflect the diverse aspects of cognitive health, ranging from temporary postsurgical changes to chronic cognitive impairments and the promotion of lifelong cognitive well-being. For a precise diagnosis and management of cognitive issues, consultation with healthcare professionals is essential. Cognitive vitality represents a holistic approach to maintaining and enhancing cognitive well-being through lifestyle choices such as mental stimulation, a balanced diet, exercise, and social engagement.

**Fig. 3:**
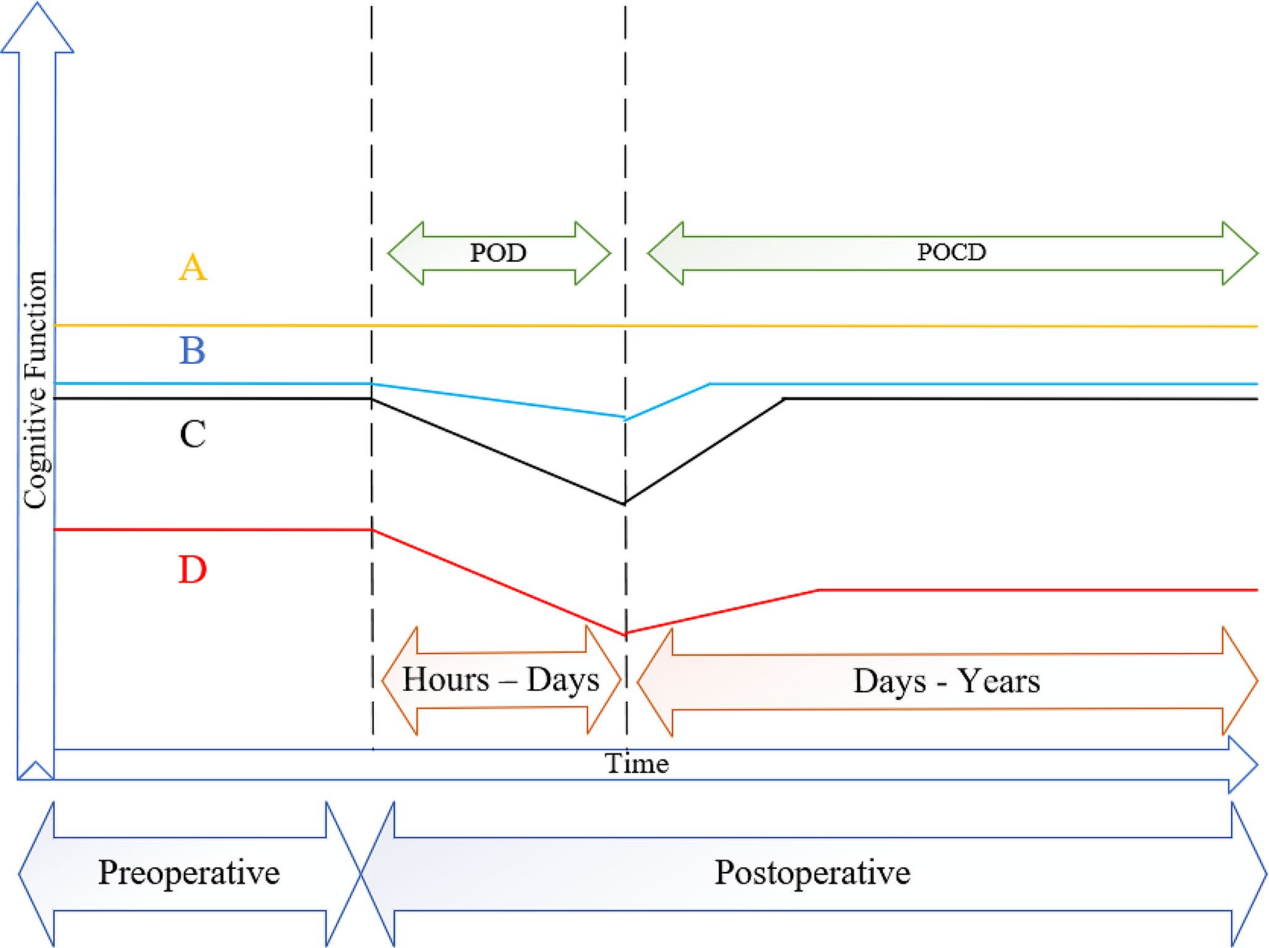
Case Examples of Trajectories of Cognitive Function Before and After Surgery

According to our findings, we provided four case examples (See Fig. 3): (**A**) a patient with a normal cognitive function who is undergoing major surgery (total knee replacement) and will have no POD and, after hospital discharge, have normal cognitive function similar to before surgery (no POCD). (**B**) a patient with a normal cognitive function who is undergoing major surgery (cardiac surgery) and will have mild POD, and after a hospital discharge, it takes 6 weeks to go back to normal cognitive function similar to before surgery (presence of reversible POCD). (**C**) a patient with normal cognitive function who is undergoing major surgery (cardiac surgery) and will have severe POD, and after a hospital discharge, it takes 10 weeks to go back to normal cognitive function similar to before surgery (presence of reversible POCD). (**D**) a patient with a major neurocognitive disorder who undegoing major surgery (hip replacement) and will have severe POD, and after hospital discharge, it takes 10 weeks to go back to cognitive function but is not similar to before surgery (presence of persistent POCD/ cognitive decline).

## Discussion

In this study, an evolutionary approach was used to analyze the concept of POCD. Various studies address POCD, focusing mainly on a specific patient group and the concept’s risk factors. A recent review also noted that the heterogeneity of previous studies may make drawing solid conclusions from the literature difficult [118]. While previous studies fell short of providing an in-depth definition of POCD, incorporating its characteristics, risk factors, outcomes, and most importantly differentiating POCD from surrogate terms commonly mistaken for, the current study provides a unified definition of POCD that can be adopted in future studies.

A few studies conducted in 1998 and afterward did adequately defined POCD [5, 12–14, 23, 36–40] most of these studies identified the reported risk factors [20, 42–48, 51, 53–63, 65, 66] and outcomes [8, 31, 32, 34, 91, 92, 94–101, 119] previously reported in our concept analysis. The problem is that most of these earlier studies were conducted without a clear theoretical framework guiding the research. Consequently, there are significant variations in the foundational principles underlying these studies. As a result, disparities have emerged concerning the precise timing, diagnosis, and even the definition of POCD. This lack of theoretical guidance has led to three potential discrepancies or issues, which, in turn, may explain the divergent incidence rates of POCD that have been reported. *First*, the three [5, 23, 24] international societies of POCD (ISPOCD) have not provided a transparent, evidence-based, well-established, and consistent definition of POCD. As a result, some studies [17, 108–110, 120–122] assessed POCD over a short duration postoperatively, while others [111, 123–125] examined POCD over a long period. To standardize the timing of assessment across studies, it is better to distinguish between POCD (occur after acute phase) and POD (occur in acute phase), as they have different pathologies and diagnostic criteria. This idea is supported by Monk et al. [6], who categorized the development of POCD as early (at hospital discharge) and late (three months after surgery). Although Monk and colleagues reported the rate of delirium and showed that the incidence of POD was higher in patients with POCD, they did not explain how they distinguished between POD and POCD. Also, POD detection-related scales such as the Richmond Agitation and Sedation Scale (RASS) or Confusion Assessment Method (CAM)-ICU were not reported. It is suggested that future studies focus on POD and report its related variables.

*Second*, because no universal agreement exists regarding tools to assess POCD [37], previous studies used different measures or scales. MMSE [16, 17, 34, 125, 126] and MoCA [17, 122] are the tests used most frequently in studies. Also, different neuropsychological tests (battery) are employed, such as Trail-Making Test A and B Digit Span Forward and Backward, Wechsler Memory Scale, and the Wechsler Adult Intelligence Scale [122, 124, 126, 127]. When using the same tools (especially tests like the MMSE) repeatedly within a short period, a potential risk of “practice effect” (PE) arises. PE involves observing pseudo-improvement in patients’ cognitive function due to repeated exposure to the same questions [13]. Various tools and scales employed in earlier research make it challenging to pinpoint the specific cognitive domains that surgery is more likely to impact. This occurrence makes the studies too heterogeneous and complex to compare. Future studies should assess at least attention, learning, and memory, which are the fundamentals of cognition and are usually lacking in POD patients [115, 128].

*Third*, functional status (including quality of life), an important aspect of differentiating mild and major NCD, and symptoms (such as pain and sleep disruption) that may be associated with POCD were usually not evaluated in previous studies [129]. This information is essential to determine if patients’ symptoms or functional status are mediators or moderators in the development of POCD or even if patients’ symptoms lie completely under NCDs. In addition, because POCD can impair quality of life [24, 33, 34, 91–93], knowing a patient’s quality of life before surgery and how it could change in the future is also important.

Other than the three previously mentioned discrepancies found in the literature, some minor methodological considerations [130] differed across the studies. Some studies [34, 42, 85, 97] did not include a control (non-surgical) group. A control group is critical because it helps in understanding the magnitude of the intervention’s (surgery’s) effect and adjusting for cognitive change caused by normal aging. Furthermore, it is suggested that patients with preexisting mild NCD be included in future studies [129] and that more advanced statistical analysis (multivariate analysis, structural equation modeling) be performed to better correlate between pre- and postoperative cognitive dysfunction with POCD.

Over the last two decades, a new term, “post-operative cognitive improvement” (POCI) emerged. POCI refers to significant improvement in cognitive function postoperatively that is not affected by a practice effect or natural variability [131]. However, which domains of cognitive function should be assessed before and after surgery is not well understood. POCI is usually reported in carotid endarterectomy (CEA) surgery [127, 15] and coronary artery bypass graft (CABG) surgery [15, 132, 133]. As discussed earlier, these studies did not include non-surgical control groups, so their findings might not be generalizable. Also, it is unclear that POCI is unaffected by practice and/or learning effects. One assumption that may be helpful for future studies is that the majority of POCI reported in patients may have been caused by symptoms such as pain, memory problems, and dyspnea; therefore, after surgery, when certain symptoms are relieved, and patients are no longer suffering, they may be able to focus (pay attention) on answering neurocognitive assessment tests. This justification was also supported by the finding of Kougias et al. [127], who showed that cognitive improvement after surgery can be seen in attention, executive function, learning, and memory. Finally, although blood biomarkers and radiological imaging [134, 135] were considered in different studies along with neurocognitive tests to detect POCD, it is unclear which findings should be considered to support this diagnosis.

### Implications for practice

Regarding the aforementioned risk factors, its advisable that strategies be developed for nurses and healthcare workers to prevent POCD: (1) screening all patients before surgery by conducting a cognitive assessment; (2) optimizing chronic conditions preoperatively appropriately; (3) applying best practices in anesthesia management (by a certified registered nurse anesthetist or anesthesiologist); (4) early screening for POD (and treating it if applicable); (5) and follow-up with patients about cognitive functioning (short- and long-term) are highly recommended.

### Limitations

Although this study has many notable strengths, two main limitations are noted. First, the literature search was limited to studies published after ISPOCD1 (1998) and written in English, so some important studies may have been missed. Second, the literature search was limited to three online databases, and gray literature was not considered in this study.

## Conclusion

POCD is a subtle-to-extensive cognitive change that affects single or multiple cognitive domains. It manifests following the acute phase of surgery, is reversible, and may last for several weeks to years. Implicitly, POCD should be assessed and detected by neuropsychological tests. POD is an acute change in cognition and should be differentiated from POCD. In certain types of surgery, patients may experience POCI after periods of POCD. However, more studies are needed to support this contention and the findings of this concept analysis.

### Electronic supplementary material

Below is the link to the electronic supplementary material.

Supplementary Material 1

## Data Availability

Not applicable.

## References

[1] Harvey PD (2019). Domains of cognition and their assessment. Dialog Clin Neurosci.

[2] Lam RW, Kennedy SH, Mclntyre RS, Khullar A (2014). Cognitive dysfunction in major depressive disorder: effects on psychosocial functioning and implications for treatment. Can J Psychiatry.

[3] McInnes K, Friesen CL, MacKenzie DE, Westwood DA, Boe SG (2017). Mild traumatic brain Injury (mTBI) and chronic cognitive impairment: a scoping review. PLoS ONE.

[4] Newman S, Stygall J, Hirani S, Shaefi S, Maze M (2007). Post-operative cognitive dysfunction after noncardiac surgery: a systematic review. Anesthesiology.

[5] Abildstrom H, Rasmussen LS, Rentowl P, Hanning CD, Rasmussen H, Kristensen PA (2000). Cognitive dysfunction 1–2 years after non-cardiac surgery in the elderly. ISPOCD group. International Study of Post-operative Cognitive Dysfunction. Acta Anaesthesiol Scand.

[6] Monk TG, Weldon BC, Garvan CW, Dede DE, van der Aa MT, Heilman KM (2008). Predictors of cognitive dysfunction after major noncardiac surgery. Anesthesiology.

[7] Tan AMY, Amoako D (2013). Post-operative cognitive dysfunction after cardiac surgery. Contin Educ Anaesth Crit Care Pain.

[8] Price CC, Garvan CW, Monk TG (2008). Type and severity of cognitive decline in older adults after noncardiac surgery. Anesthesiology.

[9] Relander K, Hietanen M, Rantanen K, Rämö J, Vento A, Saastamoinen KP (2020). Post-operative cognitive change after cardiac surgery predicts long-term cognitive outcome. Brain Behav.

[10] Folstein MF, Folstein SE, McHugh PR (1975). Mini-mental state. A practical method for grading the cognitive state of patients for the clinician. J Psychiatr Res.

[11] Nasreddine ZS, Phillips NA, Bédirian V, Charbonneau S, Whitehead V, Collin I (2005). The Montreal Cognitive Assessment, MoCA: a brief screening tool for mild cognitive impairment: Moca: a brief screening tool for MCI. J Am Geriatr Soc.

[12] Needham MJ, Webb CE, Bryden DC (2017). Post-operative cognitive dysfunction and dementia: what we need to know and do. Br J Anaesth.

[13] Liu J, Huang K, Zhu B, Zhou B, Ahmad Harb AK, Liu L (2021). Neuropsychological tests in post-operative cognitive dysfunction: methods and applications. Front Psychol.

[14] Yang X, Huang X, Li M, Jiang Y, Zhang H (2022). Identification of individuals at risk for post-operative cognitive dysfunction (POCD). Ther Adv Neurol Disord.

[15] Relander K, Hietanen M, Nuotio K, Ijäs P, Tikkala I, Saimanen E (2020). Cognitive dysfunction and mortality after carotid endarterectomy. Front Neurol.

[16] Rajaei M, Tabari M, Soltani G, Alizadeh K, Nazari A, Noroozian M (2019). Comparison between the effects of dexmedetomidine and midazolam on post-operative cognitive impairment after coronary artery bypasses graft surgery: a randomized clinical trial. J Tehran Heart Cent.

[17] Shi Y, Wang W (2019). Application of different anesthetic methods in coronary artery bypass grafting and the effect on post-operative outcome. Exp Ther Med.

[18] Steinmetz J, Rasmussen LS, Christensen IG, Lund KB, Lohse T, Ispocd Group (2008). ISPOCD Group: choice reaction time in patients with post-operative cognitive dysfunction. Acta Anaesthesiol Scand.

[19] Blokzijl F, Keus F, Houterman S, Dieperink W, van der Horst ICC, Reneman MF et al (2021) Does post-operative cognitive decline after coronary bypass affect quality of life? Open Heart. ;8(1) 10.1136/openhrt-2020-001569 PMC8070880 33888591

[20] Rundshagen I (2014). Post-operative cognitive dysfunction. Dtsch Arztebl Int.

[21] Hogan KL, Schenning KJ, Hogan KJ (2018). Trouble in mind: Healthcare informed consent, surgery, anesthesia, and the aging brain. J Leg Med (N Y).

[22] American Psychiatric Association (2022) Diagnostic and statistical manual of mental disorders (5th ed., text rev.). 10.1176/appi.books.9780890425787

[23] Moller JT, Cluitmans P, Rasmussen LS, Houx P, Rasmussen H, Canet J (1998). Long-term post-operative cognitive dysfunction in the elderly: ISPOCD1 study. Lancet.

[24] Newman MF, Kirchner JL, Phillips-Bute B, Gaver V, Grocott H, Jones RH (2001). Longitudinal assessment of neurocognitive function after coronary-artery bypass surgery. N Engl J Med.

[25] Rodgers B, Knafl K (2000) Concept analysis: an evolutionary view. Concept Dev Nursing: Found Techniques Appl. ;77–102

[26] Cheyne (2022) McCallum W. attention. In: Encyclopedia Britannica [Internet]. https://www.britannica.com/science/attention

[27] Fisher A, Kloos H (2016) Development of selective sustained attention: The role of executive functions. In J. A. Griffin, P. McCardle, & L. S. Freund (Eds.), Executive function in preschool age children: Integrating measurement, neurodevelopment, an d translational research (pp. 215 237). American Psychological Association. 10.1037/14797

[28] Cristofori I (2019) Shira Cohen-Zimerman, and Jordan Grafman. Chapter 11 - Executive Functions. In The Frontal Lobes, edited by Mark D’Esposito and Jordan H Grafman, 163:197–219. Handbook of Clinical Neurology. Elsevier. 10.1016/B978-0-12-804281-6.00011-2 10.1016/B978-0-12-804281-6.00011-2 31590731

[29] Groome D, Brace N, Edgar H, Esgate A, Pike G, Stafford T (2006). An introduction to cognitive psychology: processes and disorders.

[30] Merriam-Webster In Merriam-Webster.com dictionary. Retrieved February 17, 2023, from https://www.merriam-webster.com/dictionary/

[31] Steinmetz J, Siersma V, Kessing Lv, Rasmussen LS (2013). Is post-operative cognitive dysfunction a risk factor for dementia? A cohort follow-up study. Br J Anaesth.

[32] Evered LA, Silbert BS, Scott DA, Maruff P, Ames D (2016). Prevalence of dementia 7.5 years after coronary artery bypass graft surgery. Anesthesiology.

[33] Steinmetz J, Christensen KB, Lund T, Lohse N, Rasmussen LS, Group I (2009). Long-term consequences of post-operative cognitive dysfunction. Anesthesiology.

[34] Newman MF, Grocott HP, Mathew JP, White WD, Landolfo K, Reves JG (2001). Report of the substudy assessing the impact of neurocognitive function on quality of life 5 years after cardiac surgery. Stroke.

[35] Lezak MD (2004). Neuropsychological assessment.

[36] Fong HK, Sands LP, Leung JM (2006). The role of post-operative analgesia in delirium and cognitive decline in elderly patients: a systematic review. Anesth Analg.

[37] Krenk L, Rasmussen LS, Kehlet H (2010). New insights into the pathophysiology of post-operative cognitive dysfunction: post-operative cognitive dysfunction. Acta Anaesthesiol Scand.

[38] Rasmussen LS (2006). Post-operative cognitive dysfunction: incidence and prevention. Best Pract Res Clin Anaesthesiol.

[39] Wu CL, Hsu W, Richman JM, Raja SN (2004). Post-operative cognitive function as an outcome of regional anesthesia and analgesia. Reg Anesth Pain Med.

[40] Terrando N, Brzezinski M, Degos V, Eriksson LI, Kramer JH, Leung JM et al (2011) Perioperative cognitive decline in the aging population. Mayo Clin Proc. ;86(9):885–93 10.4065/mcp.2011.0332 PMC3257991 21878601

[41] Berger M, Terrando N, Smith SK, Browndyke JN, Newman MF, Mathew JP (2018). Neurocognitive function after cardiac surgery: from phenotypes to mechanisms. Anesthesiology.

[42] O’Gara BP, Mueller A, Gasangwa DVI, Patxot M, Shaefi S, Khabbaz K (2020). Prevention of early post-operative decline: a randomized, controlled feasibility trial of perioperative cognitive training: a randomized, controlled feasibility trial of perioperative cognitive training. Anesth Analg.

[43] Belrose JC, Noppens RR (2019). Anesthesiology and cognitive impairment: a narrative review of current clinical literature. BMC Anesthesiol.

[44] Li J (2015). Neuroprotective effects of intravenous lidocaine on early post-operative cognitive dysfunction in elderly patients following spine surgery. Med Sci Monit.

[45] Feinkohl I, Winterer G, Spies CD, Pischon T (2017). Cognitive reserve and the risk of post-operative cognitive dysfunction. Dtsch Arztebl Int.

[46] Ancelin ML, de Roquefeuil G, Ledésert B, Bonnel F, Cheminal JC, Ritchie K (2001). Exposure to anaesthetic agents, cognitive functioning and depressive symptomatology in the elderly. Br J Psychiatry.

[47] Ghoneim MM, Block RI (2012). Clinical, methodological and theoretical issues in the assessment of cognition after anaesthesia and surgery: a review: a review. Eur J Anaesthesiol.

[48] Hudetz JA, Iqbal Z, Gandhi SD, Patterson KM, Hyde TF, Reddy DM (2007). Post-operative cognitive dysfunction in older patients with a history of alcohol abuse. Anesthesiology.

[49] Hudetz JA, Patterson KM, Byrne AJ, Iqbal Z, Gandhi SD, Warltier DC (2009). A history of alcohol dependence increases the incidence and severity of post-operative cognitive dysfunction in cardiac surgical patients. Int J Environ Res Public Health.

[50] Schenning KJ, Murchison CF, Mattek NC, Kaye JA, Quinn JF (2019). Sex and genetic differences in post-operative cognitive dysfunction: a longitudinal cohort analysis. Biol Sex Differ.

[51] Silbert B, Evered L, Scott DA, McMahon S, Choong P, Ames D (2015). Preexisting cognitive impairment is associated with post-operative cognitive dysfunction after hip joint replacement surgery. Anesthesiology.

[52] Kadoi Y, Kawauchi C, Ide M, Kuroda M, Takahashi K, Saito S et al (2011) Preoperative depression is a risk factor for post-operative short-term and long-term cognitive dysfunction in patients with diabetes mellitus. J Anesth [Internet]. ;25(1):10–7. 10.1007/s00540-010-1072-5 10.1007/s00540-010-1072-5 21161290

[53] Greaves D, Psaltis PJ, Davis DHJ, Ross TJ, Ghezzi ES, Lampit A (2020). Risk factors for delirium and cognitive decline following coronary artery bypass grafting surgery: a systematic review and meta-analysis. J Am Heart Assoc.

[54] Chen H, Mo L, Hu H, Ou Y, Luo J (2021). Risk factors of post-operative delirium after cardiac surgery: a meta-analysis. J Cardiothorac Surg.

[55] Vassilaki M, Kremers WK, Machulda MM, Knopman DS, Petersen RC, Laporta ML (2022). Long-term cognitive trajectory after total joint arthroplasty. JAMA Netw Open.

[56] Lin X, Liu F, Wang B, Dong R, Sun L, Wang M (2021). Subjective cognitive decline may be associated with post-operative delirium in patients undergoing total hip replacement: the PNDABLE study. Front Aging Neurosci.

[57] Gaudet JG, Meyers PM, McKinsey JF, Lavine SD, Gray W, Mitchell E (2009). Incidence of moderate to severe cognitive dysfunction in patients treated with carotid artery stenting. Neurosurgery.

[58] Lattanzi S, Carbonari L, Pagliariccio G, Bartolini M, Cagnetti C, Viticchi G (2018). Neurocognitive functioning and cerebrovascular reactivity after carotid endarterectomy. Neurology.

[59] Heyer EJ, Mergeche JL, Wang S, Gaudet JG, Connolly ES (2015). Impact of cognitive dysfunction on survival in patients with and without statin use following carotid endarterectomy. Neurosurgery.

[60] Kulason K, Nouchi R, Hoshikawa Y, Noda M, Okada Y, Kawashima R (2017). Indication of cognitive change and associated risk factor after thoracic surgery in the elderly: a pilot study. Front Aging Neurosci.

[61] Lin X, Chen Y, Zhang P, Chen G, Zhou Y, Yu X (2020). The potential mechanism of post-operative cognitive dysfunction in older people. Exp Gerontol.

[62] Sun JH, Wu XY, Wang WJ, Jin LL (2012). Cognitive dysfunction after off-pump versus on-pump coronary artery bypass surgery: a meta-analysis. J Int Med Res.

[63] Jensen BØ, Rasmussen LS, Steinbrüchel DA (2008). Cognitive outcomes in elderly high-risk patients 1 year after off-pump versus on-pump coronary artery bypass grafting. A randomized trial. Eur J Cardiothorac Surg.

[64] Linassi F, Maran E, de Laurenzis A, Tellaroli P, Kreuzer M, Schneider G (2022). Targeted temperature management in cardiac surgery: a systematic review and meta-analysis on post-operative cognitive outcomes. Br J Anaesth.

[65] Kiabi FH, Soleimani A, Habibi MR (2019). Neuroprotective effect of low mean arterial pressure on post-operative cognitive deficit attenuated by prolonged coronary artery bypass time: a meta-analysis. Braz J Cardiovasc Surg.

[66] Larsen MH, Draegert C, Vedel AG, Holmgaard F, Siersma V, Nilsson JC (2020). Long-term survival and cognitive function according to blood pressure management during cardiac surgery. A follow-up. Acta Anaesthesiol Scand.

[67] Feng X, Hu J, Hua F, Zhang J, Zhang L, Xu G (2020). The correlation of intraoperative hypotension and post-operative cognitive impairment: a meta-analysis of randomized controlled trials. BMC Anesthesiol.

[68] Mason SE, Noel-Storr A, Ritchie CW (2010). The impact of general and regional anesthesia on the incidence of post-operative cognitive dysfunction and post-operative delirium: a systematic review with meta-analysis. J Alzheimers Dis.

[69] Bhushan S, Huang X, Duan Y, Xiao Z (2022). The impact of regional versus general anesthesia on post-operative neurocognitive outcomes in elderly patients undergoing hip fracture surgery: a systematic review and meta-analysis. Int J Surg.

[70] Negrini D, Wu A, Oba A, Harnke B, Ciancio N, Krause M (2022). Incidence of post-operative cognitive dysfunction following inhalational vs total intravenous general anesthesia: a systematic review and meta-analysis. Neuropsychiatr Dis Treat.

[71] Pang QY, Duan LP, Jiang Y, Liu HL (2021). Effects of inhalation and propofol anaesthesia on post-operative cognitive dysfunction in elderly noncardiac surgical patients: a systematic review and meta-analysis: a systematic review and meta-analysis. Med (Baltim).

[72] Zhang Y, Shan GJ, Zhang YX, Cao SJ, Zhu SN, Li HJ, Ma D, Wang DX, First Study of Perioperative Organ Protection (SPOP1) investigators (2018). Propofol compared with sevoflurane general anaesthesia is associated with decreased delayed neurocognitive recovery in older adults. Br J Anaesth.

[73] Govêia CS, de Miranda DB, Oliveira LV, de Praxedes B, Moreira FB, Guimarães LG (2021). Dexmedetomidine reduces post-operative cognitive and behavioral dysfunction in adults submitted to general anesthesia for non-cardiac surgery: meta-analysis of randomized clinical trials. Braz J Anesthesiol.

[74] Yu H, Kang H, Fan J, Cao G, Liu B (2022). Influence of dexmedetomidine on post-operative cognitive dysfunction in the elderly: a meta-analysis of randomized controlled trials. Brain Behav.

[75] Li J, Yin Q, Xun X, He J, Yu D, Wang Z (2021). The effect of intraoperative dexmedetomidine on cognitive dysfunction after surgery: a updated meta-analysis. J Cardiothorac Surg.

[76] Hovaguimian F, Tschopp C, Beck-Schimmer B, Puhan M (2018). Intraoperative ketamine administration to prevent delirium or post-operative cognitive dysfunction: a systematic review and meta-analysis. Acta Anaesthesiol Scand.

[77] Bocskai T, Kovács M, Szakács Z, Gede N, Hegyi P, Varga G (2020). Is the bispectral index monitoring protective against post-operative cognitive decline? A systematic review with meta-analysis. PLoS ONE.

[78] Lu X, Jin X, Yang S, Xia Y (2018). The correlation of the depth of anesthesia and post-operative cognitive impairment: a meta-analysis based on randomized controlled trials. J Clin Anesth.

[79] Ding L, Chen DX, Li Q (2020). Effects of electroencephalography and regional cerebral oxygen saturation monitoring on perioperative neurocognitive disorders: a systematic review and meta-analysis. BMC Anesthesiol.

[80] Seven S, Ceylan İlkay, Kaymak D, Kara AG, Erden V (2022) The effect of type 2 diabetes mellitus on early postoperative cognitive functions. J Surg Med [Internet]. May 1 [cited 2023 Dec. 9];6(5):552-6. https://jsurgmed.com/article/view/947765

[81] Lachmann G, Feinkohl I, Borchers F, Ottens TH, Nathoe HM, Sauer AM (2018). Diabetes, but not hypertension and obesity, is associated with post-operative cognitive dysfunction. Dement Geriatr Cogn Disord.

[82] Feinkohl I, Winterer G, Pischon T (2017) Diabetes is associated with risk of post-operative cognitive dysfunction: A meta-analysis. Diabetes Metab Res Rev [Internet]. ;33(5):e2884. https://onlinelibrary.wiley.com/doi/abs/10.1002/dmrr.2884 10.1002/dmrr.2884 28063267

[83] Kadoi Y, Saito S, Fujita N, Goto F (2005). Risk factors for cognitive dysfunction after coronary artery bypass graft surgery in patients with type 2 diabetes. J Thorac Cardiovasc Surg.

[84] Feinkohl I, Winterer G, Pischon T (2017). Hypertension and risk of post-operative cognitive dysfunction (POCD): a systematic review and meta-analysis. Clin Pract Epidemiol Ment Health.

[85] Puskas F, Grocott HP, White WD, Mathew JP, Newman MF, Bar-Yosef S (2007). Intraoperative hyperglycemia and cognitive decline after CABG. Ann Thorac Surg.

[86] Butterworth J, Wagenknecht LE, Legault C, Zaccaro DJ, Kon ND, Hammon JW Jr et al (2005) Attempted control of hyperglycemia during cardiopulmonary bypass fails to improve neurologic or neurobehavioral outcomes in patients without diabetes mellitus undergoing coronary artery bypass grafting. J Thorac Cardiovasc Surg. ;130(5):1319.e1-1319.e9 10.1016/j.jtcvs.2005.02.049 16256784

[87] He X, Long G, Quan C, Zhang B, Chen J, Ouyang W (2019). Insulin Resistance predicts post-operative cognitive dysfunction in elderly gastrointestinal patients. Front Aging Neurosci.

[88] Deiner S, Silverstein JH (2009). Post-operative delirium and cognitive dysfunction. Br J Anaesth.

[89] Goldberg TE, Chen C, Wang Y, Jung E, Swanson A, Ing C (2020). Association of delirium with long-term cognitive decline: a meta-analysis: a meta-analysis. JAMA Neurol.

[90] Daiello LA, Racine AM, Yun Gou R, Marcantonio ER, Xie Z, Kunze LJ (2019). Post-operative delirium and post-operative cognitive dysfunction: overlap and divergence: overlap and divergence. Anesthesiology.

[91] Mashour GA, Woodrum DT, Avidan MS (2015). Neurological complications of surgery and anaesthesia. Br J Anaesth.

[92] Gold S, Forryan S (2019). Post-operative cognitive decline: a current problem with a difficult future. Tren Anaesth Crit Care.

[93] Phillips-Bute B, Mathew JP, Blumenthal JA, Grocott HP, Laskowitz DT, Jones RH (2006). Association of neurocognitive function and quality of life 1 year after coronary artery bypass graft (CABG) surgery. Psychosom Med.

[94] Schenning KJ, Murchison CF, Mattek NC, Silbert LC, Kaye JA, Quinn JF (2016). Surgery is associated with ventricular enlargement as well as cognitive and functional decline. Alzheimers Dement.

[95] Inouye SK, Marcantonio ER, Kosar CM, Tommet D, Schmitt EM, Travison TG (2016). The short-term and long-term relationship between delirium and cognitive trajectory in older surgical patients. Alzheimers Dement.

[96] Berger M, Burke J, Eckenhoff R, Mathew J (2014). Alzheimer’s disease, anesthesia, and surgery: a clinically focused review. J Cardiothorac Vasc Anesth.

[97] Lundström M, Edlund A, Bucht G, Karlsson S, Gustafson Y (2003). Dementia after delirium in patients with femoral neck fractures. J Am Geriatr Soc.

[98] Pietzsch M, Weber SA, Winterer G, Lammers-Lietz F, Borchers F, Hadzidiakos D (2021). A model-based estimation of annual long-term care costs in Germany following post-operative cognitive dysfunction (POCD) in elderly patients. J Public Health Int.

[99] Boone MD, Sites B, von Recklinghausen FM, Mueller A, Taenzer AH, Shaefi S (2020). Economic burden of post-operative neurocognitive disorders among US Medicare patients. JAMA Netw Open.

[100] Schmitt EM, Saczynski JS, Kosar CM, Jones RN, Alsop DC, Fong TG (2015). The successful aging after elective surgery (SAGES) study: cohort description and data quality procedures. J Am Geriatr Soc.

[101] Kimchi EY, Hshieh TT, Guo R, Wong B, O’Connor M, Marcantonio ER (2017). Consensus approaches to identify incident dementia in cohort studies: systematic review and approach in the successful aging after elective surgery study. J Am Med Dir Assoc.

[102] Whitlock EL, Vannucci A, Avidan MS (2011). Postoperative delirium. Minerva Anestesiol.

[103] Schubert M, Schürch R, Boettger S, Garcia Nuñez D, Schwarz U, Bettex D et al (2018) A hospital-wide evaluation of delirium prevalence and outcomes in acute care patients - a cohort study. BMC Health Serv Res. ;18(1) 10.1186/s12913-018-3345-x PMC6045819 30005646

[104] Ho MH, Nealon J, Igwe E, Traynor V, Chang HCR, Chen KH (2021). Post-operative delirium in older patients: a systematic review of assessment and incidence of post-operative delirium. Worldviews Evid Based Nurs.

[105] Gale SA, Acar D, Daffner KR (2018). Dement Am J Med.

[106] Arvanitakis Z, Shah RC, Bennett DA (2019). Diagnosis and management of dementia. Rev JAMA.

[107] Smits LL, van Harten AC, Pijnenburg YAL, Koedam ELGE, Bouwman FH, Sistermans N (2015). Trajectories of cognitive decline in different types of dementia. Psychol Med.

[108] Petersen RC (2004). Mild cognitive impairment as a diagnostic entity. J Intern Med.

[109] Petersen RC, Lopez O, Armstrong MJ, Getchius TSD, Ganguli M, Gloss D (2018). Practice guideline update summary: mild cognitive impairment: report of the Guideline Development, Dissemination, and implementation Subcommittee of the American Academy of Neurology. Neurology.

[110] Domínguez-Chávez CJ, Murrock CJ, Salazar-González BC (2019). Mild cognitive impairment: a concept analysis. Nurs Forum.

[111] Jongsiriyanyong S, Limpawattana P (2018). Mild cognitive impairment in clinical practice: a review article. Am J Alzheimers Dis Other Demen.

[112] Tangalos EG, Petersen RC (2018). Mild cognitive impairment in geriatrics. Clin Geriatr Med.

[113] Harerimana B The Concept of Cognitive Vitality: Analysis and Clinical and Research Implications. 2020–30

[114] McDonough IM, Haber S, Bischof GN, Park DC (2015). The Synapse Project: Engagement in mentally challenging activities enhances neural efficiency. Restor Neurol Neurosci.

[115] Gow AJ, Whiteman MC, Pattie A, Whalley L, Starr J, Deary IJ (2005). Lifetime intellectual function and satisfaction with life in old age: longitudinal cohort study. BMJ.

[116] Farias ST, Mungas D, Reed BR, Harvey D, DeCarli C (2009). Progression of mild cognitive impairment to dementia in clinic- vs community-based cohorts. Arch Neurol.

[117] Soenarto RF, Mansjoer A, Amir N, Aprianti M, Perdana A (2018) Cardiopulmonary bypass alone does not cause post-operative cognitive dysfunction following open heart surgery. Anesth Pain Med. ;8(6) 10.5812/aapm.83610 PMC6347738 30719417

[118] Borchers F, Spies CD, Feinkohl I, Brockhaus WR, Kraft A, Kozma P (2021). Methodology of measuring post-operative cognitive dysfunction: a systematic review. Br J Anaesth.

[119] Hovens IB, Schoemaker RG, van der Zee EA, Heineman E, Izaks GJ, van Leeuwen BL (2012). Thinking through post-operative cognitive dysfunction: how to bridge the gap between clinical and pre-clinical perspectives. Brain Behav Immun.

[120] Soliman R, Saad D, Abukhudair W, Abdeldayem S (2022). The neurocognitive outcomes of hemodilution in adult patients undergoing coronary artery bypass grafting using cardiopulmonary bypass. Ann Card Anaesth.

[121] Zhao Q, Gao R, Liu C, Chen H, Zhang X, Guan J (2021). Dynamic change of lymphocyte-to-monocyte is associated with the occurrence of POCD after cardiovascular surgery: a prospective observational study. Front Behav Neurosci.

[122] Nurcahyo WI, Arifin A, Primatika AD, Muttaqin Z, Elfira Boom C, Harahap MS (2021). An association between C-reactive protein levels and the occurrence of cognitive dysfunction after heart valve replacement. Vasc Health Risk Manag.

[123] Yin YQ, Luo AL, Guo XY, Li LH, Huang YG (2007). Post-operative neuropsychological change and its underlying mechanism in patients undergoing coronary artery bypass grafting. Chin Med J (Engl).

[124] Ottens TH, Dieleman JM, Sauër AMC, Peelen LM, Nierich AP, de Groot WJ (2014). Effects of dexamethasone on cognitive decline after cardiac surgery: a randomized clinical trial: a randomized clinical trial. Anesthesiology.

[125] Mohandas BS, Jagadeesh AM, Vikram SB (2013). Impact of monitoring cerebral oxygen saturation on the outcome of patients undergoing open heart surgery. Ann Card Anaesth.

[126] Ganguly G, Dixit V, Patrikar S, Venkatraman R, Gorthi SP, Tiwari N (2015). Carbon dioxide insufflation and neurocognitive outcome of open heart surgery. Asian Cardiovasc Thorac Ann.

[127] Kougias P, Collins R, Pastorek N, Sharath S, Barshes NR, McCulloch K (2015). Comparison of domain-specific cognitive function after carotid endarterectomy and stenting. J Vasc Surg.

[128] Devinney MJ, Mathew JP, Berger M (2018). Post-operative delirium and post-operative cognitive dysfunction: two sides of the same coin?. Anesthesiology.

[129] Nadelson MR, Sanders RD, Avidan MS (2014). Perioperative cognitive trajectory in adults. Br J Anaesth.

[130] Funder KS, Steinmetz J, Rasmussen LS (2010). Methodological issues of post-operative cognitive dysfunction research. Semin Cardiothorac Vasc Anesth.

[131] Arias F, Sibille KT, Price CC (2019) Post-operative cognitive improvement. The Perioperative Neurocognitive disorders. Cambridge University Press, pp 34–47

[132] Kennedy ED, Choy KCC, Alston RP, Chen S, Farhan-Alanie MMH, Anderson J (2013). Cognitive outcome after on- and off-pump coronary artery bypass grafting surgery: a systematic review and meta-analysis. J Cardiothorac Vasc Anesth.

[133] Cormack F, Shipolini A, Awad WI, Richardson C, McCormack DJ, Colleoni L (2012). A meta-analysis of cognitive outcome following coronary artery bypass graft surgery. Neurosci Biobehav Rev.

[134] Androsova G, Krause R, Winterer G, Schneider R (2015) Biomarkers of post-operative delirium and cognitive dysfunction. Front Aging Neurosci. ;7 10.3389/fnagi.2015.00112 PMC4460425 26106326

[135] Sun X, Lindsay J, Monsein LH, Hill PC, Corso PJ (2012). Silent brain injury after cardiac surgery: a review: cognitive dysfunction and magnetic resonance imaging diffusion-weighted imaging findings. J Am Coll Cardiol.

