# Supplementary material for: Postoperative cognitive dysfunction: a concept analysis
Source: Aging Clin Exp Res. 2024 Jun 21;36(1):133. doi: 10.1007/s40520-024-02779-7 (PMC11189971; doi:10.1007/s40520-024-02779-7)
Supplement: Supplementary file 1 — Supplementary Material 1 [file 40520_2024_2779_MOESM1_ESM.docx]

| Appendix 1: Summary of Included Studies | | | | | | |
| --- | --- | --- | --- | --- | --- | --- |
| Author (Year)/ Country | Study Design/ Sample Size | Population | Key notes | Attributes | Antecedents | Consequences |
| Newman (2007) / UK (4) | Systematic review | Noncardiac surgery | Indicates studies that don’t use control groups cannot control variables such as learning effect, thus finding a lower prevalence of POCD | Suggest the role of pain and medication on early postoperative period cognitive function | NA | NA |
| Abildstrom (2000) / multinational (5) | Cohort / 366 | > 60 years  Noncardiac surgery | Study found no relation between POCD in 3 months and 1-2 years with long-term POCD | Study found POCD to be mostly reversible but also can become persistent in 1% of patients | Age, early POCD and infection during 3 months postoperative were related to long-term cognitive impairment | NA |
| Monk (2008) / USA (6) | Cohort / 1,064 | Noncardiac surgery | While being common in all adults, POCD only increases the risk of long-term cognitive impairment for patients older than 60 years | Measurement of POCD was done at discharge from hospital and 3 months postoperative | POCD at 3 months was related to higher age, lower level of education, history of cerebral vascular injury and POCD at discharge | POCD at discharge and 3 months was related to higher mortality rate |
| Price (2008) / USA (8) | Case- control / 417 | Noncardiac surgery | Patients with executive or both executive and memory impairment had more deficits in instrumental activities of daily living | Patients developed cognitive function impairment in memory, executive function or both domains postoperative | NA | POCD was related to greater impairment in instrumental activities of daily living |
| Needham (2017) / UK (12) | Narrative review | NA | Study suggests a lack of accurate information for patients and also an agreed definition and assessment of POCD | Study characterizes POCD to begin between 7 days and one year postoperative | Study discusses several risk factors | NA |
| Liu (2021) / China (13) | Narrative review | NA | Study suggests the use of a set of neurocognitive tools that have fewer overlap for the diagnosis of POCD | NA | NA | NA |
| Yang (2022) / China (14) | Narrative review | NA | Study suggests risk assessment to be done at hospital admission, during surgery and postoperatively and if POCD remains at discharge, follow-up be done | NA | study discusses preoperative, intraoperative, and postoperative risk factors of POCD | NA |
| Steinmetz (2008) / Multinational (18) | Cohort / 1,083 | > 60 years  Various | Although choice reaction time showed to be significantly longer in patients with POCD it showed a low positive predictive value | NA | NA | Patients with POCD showed longer reaction times |
| Rundshagen (2014) / Germany (20) | Narrative review | NA | Study reviews definition, incidence, measuring tools, pathogenesis, risk factors and prevention and treatments of POCD | Defines POCD as a new cognitive impairment happening postoperative | Study discusses several risk factors | Study discusses the role of POCD in poorer recovery, higher mortality and increased use of social financial assistance |
| Moller (1998)/ Multinational  (23) | Prospective Cohort/ 1,539 | ≥ 60 years  Major non-cardiac surgery | out of the risk factors measured only higher age was related to late (3 months) POCD | Measurement of POCD was done at 1 week and 3 months. | Higher age and duration of anesthesia, lower education, second operation, infections and respiratory complications | POCD is related to decreased activity of daily living. |
| Newman (2001) / USA (24) | Cohort/ 261 | Elective CABG | High prevalence of POCD after CABG (42% after 5 years) | Cognitive dysfunction could last 5 years.  Change of 1 SD or more in any domain was evaluated | Cognitive decline at discharge, older age, lower education, and higher composite baseline cognitive score | NA |
| Fong (2006) / USA (30) | Systematic review | NA | No difference between parenteral vs epidural anesthesia | NA | Meperidine was the only opioid found to be related to POCD. | NA |
| Krenk (2010) / Denmark (31) | Narrative review | NA | Intervention, hospital associated, preoperative and postoperative factors are related to POCD | POCD is more subtle and long lasting than delirium | Age, pre-existing disease, low level of education and cognitive function, minimal invasive surgery, pain control, early discharge, pharmacological sleep improvement and reduction in nighttime noise | NA |
| Rasmussen (2006) / Denmark (32) | Narrative review | NA | Study suggest data from control groups to be beneficial because it can help to interpret variability in results | POCD is a subtle change in cognitive function needing neuropsychological testing to be detected | Several risk factors were discussed | NA |
| Christopher (2004) / USA (33) | Systematic review | NA | Many concerns with assessment of cognitive function and tests in studies. | NA | No difference between General vs neuraxial anesthesia in POCD | NA |
| Terrando (2011) / USA (34) | Review | NA | Study proposes a road map for further investigations covering different aspects of POCD | POCD is more subtle and prolonged than POD. | POCD is a more than expected decline in different domains of cognition. | NA |
| Steinmetz (2009)/ Denmark (35) | Cohort/701 | Non-cardiac surgery/ | No association between mortality and POCD 1 week after surgery, but significant for POCD at 3 months. | Measurement done at 1 week and 3 months. | NA | Increased mortality, risk of leaving labor market, prevalence of receiving social transfer payments. |
| Steinmetz (2013) / Denmark (36) | Cohort/ 686 | Non-cardiac surgery | No association between POCD and dementia or depression after 11 years was found. | POCD is largely reversible | NA | NA |
| Berger (2018) / USA (37) | Narrative review | NA | Study proposes development of “bundle” protocols to tackle POCD. | POCD rate declines over time.  Cognitive decline can occur in single or multiple domains | Several risk factors discussed | NA |
| Evered (2016) / Australia (38) | Cohort/ 326(276) | Elective CABG | See attributes column | POCD in 32.8% of patients after 7.5 years | Early POCD is related to long term POCD | Increased risk of mortality |
| Newman (2001) / USA (39) | Cohort/ 261 | Elective CABG | 42% of patients had a cognitive decline of at least 1 SD in one domain after 5 years. | Neurocognitive testing was done 7 days, 6 weeks, 6 months and 5 years postoperative | NA | Lower 5-year cognitive function correlates with lower quality of life score. |
| O’Gara (2020)/ Israel (40) | RCT/45 | 60-90 years  Cardiac surgery | Overall incidence of POCD at discharge was 44% | NA | Cognitive training couldn’t reduce POCD incidence. | NA |
| Belrose (2019) / Canada (41) | Narrative review | NA | Reviews the role of Alzheimer’s disease and other similar diseases in POCD incidence and underlying mechanism | Defines POCD from expected recovery (30 days) to 12 months postoperative and mentions a mild and major subtype | Several risk factors discussed | NA |
| Li (2015)/ China (42) | RCT/ 80 | > 65 years  spine surgery | Lidocaine suppresses IL-6, S100b, and NSE thus may be an effective neuroprotective agent in POCD prevention | POCD was measured 3 days postoperative | Lidocaine administration during anaesthesia resulted in better cognitive function than control postoperative 3 | NA |
| Feinkohl (2017) / Germany (43) | Systematic review and meta-analysis / 5,104 | NA | See antecedents column | NA | Lower education was related to higher POCD incidence | NA |
| Ancelin (2001)/ France (44) | Cohort/ 140 | > 64 years  Programmed orthopedic surgery | Domains of cognition were deteriorated differently, secondary and implicit memory, visuospatial and linguistic were affected more. | Cognitive function was measured at 9 days and at 3 months postop. | Patients with lower education, higher age, preoperative depression and history of cognitive deterioration showed highest cognitive decline. | NA |
| Ghoneim (2012) / USA (45) | Narrative review | NA | Study discusses benefits of dichotomization vs. continuous measurement of cognitive decline | Proposes testing within first week postop | Several risk factors discussed (age, education, vascular disease, type of anesthesia and surgery) | NA |
| Hudetz (2007) / USA (46) | Cohort/ 56 | 55-81 years  Various surgeries | Decline in visuospatial recent memory was greater than executive function. | NA | Alcohol abuse related POCD was associated with history of diabetes mellitus (DM), depression, ICU or ward administration and high blood loss. | NA |
| Hudetz (2009) / USA (47) | Cohort / 103 | > 55  Cardiac surgery | Preoperative alcohol induced cognitive function doesn’t affect postoperative cognitive function. | POCD was measured 3 and 7 days postoperative | Alcohol abuse requiring hospitalization is associated with POCD. | NA |
| Schenning (2019) / USA (48) | Retrospective analysis of multiple cohorts/ 1,033 | NR | Differences between sex and POCD incidence is related to APOE4 allele. | NA | Men with APOE4 allele had more POCD incidence and severity. | NA |
| Silbert (2015) / Australia (49) | Prospective observational trial/ 300 | ≥ 60 years  Elective total hip replacement | PreCI is a good predictor of POCD | Measurement was done at 7 days, 3 and 12 months after. | Preexisting cognitive impairment is associated with POCD. | NA |
| Farias (2009) / USA (50) | Cohort / 111 | No surgery | Study defines MCI as a state of cognitive function transitioning from normal cognition to Alzheimer’s disease and found the greater functional impairment is related to higher chance of developing dementia in patients with MCI. (surrogate term : MCI) | | | |
| Kadoi (2011) / Japan (51) | Cohort / 100 | CABG | Preoperative depression is associated with short-term and long-term POCD | Measurement was done at 7 days and 6 months after. | Age, hypertension, depression, jugular venous oxygen saturation, ascending aorta atherosclerosis, diabetic retinopathy,  insulin therapy and HbA1c were associated with POCD | NA |
| Greaves (2020) / Australia (52) | Systemaic review and meta-analysis / 60,479 | CABG | Identifying modifiable risk factor of POCD can reduce incidence | NA | History of stroke, hypertension, DM, older age and higher EuroSCORE to acute-(until 1months postop), preoperative depression and higher cognitive test scores to mid-term (1-6 months postop), while no significant risk factors to long-term (12-15 months postop) cognitive decline were related | NA |
| Chen (2021)/ China (53) | Systematic review and meta-analysis/ 13,286 | Cardiac surgery | Study defines delirium as a disturbance in attention and awareness with a fluctuating form, with an additional disturbance in cognition all of which can’t be better explained by another neurocognitive disorder. Delirium is classified in 3 subtypes: hypoactive, hyperactive, and mixed form. | | | |
| Vassilaki (2022) / USA (54) | Cohort / 5,550 | Total hip or knee arthroplasty | Annual Cognitive decline of patients with and without joint arthroplasty didn’t differ significantly, except for patients > 80 years with knee arthroplasty. | NA | Patients older than 80 years undergoing total knee arthroplasty showed slightly faster cognitive decline. | NA |
| Lin (2021) / China (55) | Cohort / 214 | > 40 years  Total hip replacement | Study found that subjective cognitive decline (i.e. cognitive decline of an individual has declined compared to previous stat while neuropsychological test is normal) is a risk factor for POD. | | | |
| Gaudet (2009) / USA (56) | Cohort/ 47 | elective carotid artery stenting | carotid artery stenting is associated with decline of cognitive function. | Measurement was done 1 day and 1 month after. Most patients improved between measurements. | Statin medication reduces POCD. POCD is independent of age or previous neurological events. | NA |
| Lattanzi (2018) / Italy (57) | Cohort / 137 | carotid endarterectomy | Carotid endarterectomy improved cognitive function 6 months after surgery in patients with a history of TIA and ipsilateral high-grade ICA stenosis. | NA | See key notes | NA |
| Heyer (2015) / USA (58) | Post hoc analysis of a prospective observational study / 558 | ≥ 60 years Elective carotid endarterectomy | Early cognitive dysfunction is an important factor for overall health, comorbidity, and neurological vulnerability. | NA | See key notes | Early cognitive dysfunction is associated with lower survival in patients without using statin medication. |
| Kulason (2017)/ Japan (59) | Cohort / 12 | Major thoracic surgery | Preoperative mental wellbeing is a possible predictor of POCD. | Cognitive decline is significant in attention and psychomotor function domains | Longer anaesthesia is related to decline in attention and working memory postoperative.  Age and gender are not related to POCD. | NA |
| Lin (2020) / China (60) | Narrative review | NA | Study discusses the role of peripheral (e.g. inflammation and immune activation), central (e.g. neurons and neurotoxicity), microbiome and brain regions in POCD pathophysiology | NA | See key notes | NA |
| Sun (2012) / China (61) | Systematic review and meta-analysis/ 2,326 | CABG | Although significantly different at early stage, no difference between of-pump and conventional CABG related to POCD incidence was found at 6 and 12 months postop. | NA | Off-pump coronary artery bypass compared to conventional CABG has lower risk of POCD measured 1-2 weeks and 3 months postop. | NA |
| Jensen (2008) / Denmark (62) | Sub-study of RCT / 120 | ≥ 55 years  Elective or subacute CABG | No significant difference between on- and off-pump surgery in 1-year cognitive function | NA | See key notes | NA |
| Linassi (2022) / Italy (63) | Systematic review and meta-analysis / 9,609 | Cardiac surgery | Temperature, mean arterial pressure (MAP), and surgery type were no related to POCD | NA | Neurocognitive decline was lower in normothermic and targeted MAP > 70 cardiopulmonary bypass surgeries | NA |
| Kiabi (2019) / Iran (64) | Systematic review and meta-analysis / 731 | Cardiopulmonary bypass surgery | Low MAP surgery doesn’t reduce POCD incidence | NA | Lower surgery time, and older age in low MAP surgeries is related to POCD | NA |
| Larsen (2020)/ Denmark (65) | RCT/ 197 | Cardiopulmonary bypass surgery | No significant difference in POCD between patients with high or low target blood pressure during surgery | NA | See key notes | NA |
| Feng (2020) / China (66) | Systematic review and meta-analysis / 701 | Various | No significant relation between intraoperative hypotension and POCD | NA | See key notes | NA |
| Mason (2010) UK (67) | Systematic review and meta-analysis / | Various | No significant relation between type of anaesthesia and POCD was found | NA | See key notes | NA |
| Bhushan (2022) / China (68) | Systematic review and meta-analysis / 3,555 | > 65 years  Hip fracture surgery | No significant difference between general and regional anaesthesia for the incidence of POD / POCD observed | NA | See key notes | NA |
| Negrini (2022) / Brazil (69) | Systematic review and meta-analysis / 3,390 | Various | Study found a heterogeneity of criteria for POCD incidence 1 to 12 months postoperative in papers. | NA | At 30 days postoperative total intravenous anaesthesia is related to lower POCD rates compared to inhalational anaesthesia | NA |
| Zhang (2018)/ China (70) | RCT/ 387 | Cancer surgery | Anesthesia based on propofol compared to sevoflurane is associated with reduced risk of delayed cognitive deficits after cancer surgery. | Neurocognitive assessment was done 1 week postoperative for patients and controls and diagnosis was done using ISPOCD1 definition | Propofol compared to sevoflurane has a lower risk of delayed cognitive recovery | NA |
| Xu (2012) / China (71) | Systematic review and meta-analysis / 1,854 | Non-cardiac surgery | See antecedents column | NA | Propofol compared to inhalational anaesthesia is related to lower POCD in 2-6 days postop. | NA |
| Pang (2021) / China (72) | Meta-analysis / 1,626 | > 60 years  Various | See antecedents column | NA | Perioperative administration of dexmedetomidine in elderly reduces POCD incidence compared to placebo | NA |
| Li (2021) / China (73) | Meta-analysis / 2,902 | Various | See antecedents column | NA | Dexmedetomidine reduces POCD incidence on the 1^st^, 3^rd^, and 7^th^ postoperative day and improves postoperative MMSE score | NA |
| Govêia (2021) / Brazil (74) | Meta-analysis / 2,183 | Non- cardiac | There is no recommended timing for dexmedetomidine administration (e.g. Induction, intraoperative, postop) | NA | Dexmedetomidine administration reduces chance of postoperative cognitive and behavioral dysfunction | NA |
| Hovaguimian (2018) / Switzerland (75) | Systematic review and meta-analysis / 163 | Various | While effective against POCD, intraoperative ketamine effect in preventing POD is insignificant | NA | Ketamine administration intraoperative is protective against POCD incidence | NA |
| Bocskai (2020) / Hungary (76) | Systematic review and meta-analysis / 1985 | Various | Although use of Bispectral index monitoring (BIS) was protective against POCD at 12 weeks but wasn’t found significant at 1 week postop. | NA | Use of BIS is associated with lower POCD incidence at 12 weeks postop. | NA |
| Lu (2018) / China (77) | Meta-analysis / 340 | Various | No difference between low and high BIS anaesthesia | NA | See key notes | NA |
| Ding (2020) / China (78) | Systematic review and meta-analysis / 6,356 | Various | Use of intraoperative EEG- monitoring was insignificant in early POCD prevention but significant up to 3 months | NA | Intraoperative regional cerebral oxygen saturation and EEG- monitoring of anaesthesia reduces POCD incidence. | NA |
| Lachmann (2018) / Germany (79) | Secondary analysis of 3 RCTs / 1,034 | Various | Obesity or hypertension were not associated with POCD | NA | DM was associated to POCD incidence  (1.84-fold) | NA |
| Feinkohl (2017) / Germany (80) | Systematic review and meta-analysis / 2,518 | Various | See antecedents column | NA | DM was associated with POCD incidence (1.26-fold) | NA |
| Kadoi (2005) / Japan (81) | Cohort / 200 | Elective CABG | Hypertension, reduced SvO2, and ascending aorta atherosclerosis was related to early ( 7 days postop) POCD.  Insulin therapy, diabetic retinopathy, and HbA1c was related to late (6 months postop) POCD. | NA | Higher age, type 2 DM, hypertension, lower jugular venous oxygen saturation, ascending aorta atherosclerosis, diabetic retinopathy and HbA1c and insulin therapy were associated to POCD | NA |
| Seven (2022) / Turkey (82) | Prospective case-control / 50 | < 70 years  Elective laparoscopic cholecystectomy | Patients with diabetes showed lower MMSE and MoCA postop | POCD was measured at 4^th^ and 24^th^ hours postop | DM is related to higher POCD incidence | NA |
| Soenarto (2018) / Indonesia (83) | Cohort / 54 | Open heart surgery | Education, DM, duration of cardiopulmonary bypass and cross-clamp were not related to POCD | NA | Patients older than 65 years had higher chance of cognitive decline | NA |
| Feinkohl (2017) / Germany (84) | Systematic review and meta-analysis / 4,317 | Various | Hypertension is not associated with POCD | NA | See key notes | NA |
| Puskas (2007) / USA (85) | Cohort / 525 | CABG | Although related in nondiabetic patients, hyperglycemia in diabetic patients is not related to POCD | Cognitive function measured 6 weeks postop | In nondiabetic patients, intraoperative hyperglycemia is related to POCD incidence | NA |
| Butterworth (2005) / USA (86) | Cohort / 381 | 35 – 80 years  CABG | Intraoperative Control of hyperglycemia doesn’t affect short- or long- term outcomes | Measurement done at 4-8 days, 6 weeks and 6 months postop | See key notes | NA |
| He (2019) / China (87) | Cohort / 124 | > 60 years  Gastrointestinal surgery | Patients with POCD performed worse in Visuospatial Memory, Trail Making Test, and Digit Span Test. | Measurement was done 7 days postop | Insulin resistance is associated with POCD | NA |
| Daiello (2019) / USA (88) | Cohort / 560 | > 70 years  Various | POD and POCD are potentially different neurocognitive events because POCD was present more in patients without POD at each measurement | Measurement was done 1, 2 and 6 months postop | POD only in the first month postoperative is related to POCD | NA |
| Deiner (2009) / USA (89) | Narrative review | NA | Study reviews definition, pathophysiology, aetiology, treatment and prevention of POD and POCD | Defines POCD as a temporary deterioration in cognition, affecting one or more domains of cognition. | Several risk factors were discussed | NA |
| Goldberg (2020) / USA (90) | Systematic review and meta-analysis / 4,317 | NA | See antecedents column | NA | Long- term cognitive decline was related to POD | NA |
| Mashour (2015) / USA (91) | Narrative review | NA | Study argues the need for matched control groups to better understand the underlying reasons resulting in POCD incidence | NA | Several risk factors were discussed | Several postoperative complications were briefly reviewed |
| Gold (2019) / UK (92) | Narrative review | NA | Study believes methodological problems (e.g., the lack of universal definition and measurement of POCD) impeding research and meta- analysis to be done to a comprehension | Defines POCD as a postoperative relative decline in different domains of cognition, that while missable, can affect daily functions | Several risk factors were discussed | NA |
| Phillips-Bute (2006) / USA (93) | Cohort / 732 | CABG | Study found no single domain of cognitive function to be related to quality of life (QOL) but found the effect to be composite of the different domains | Measurement was done 6 weeks and 1 year after surgery | NA | POCD is associated with decline in QOL (e.g., lower functional capacity and activities of daily living, more depression and self-reported mental difficulties) |
| Schenning (2016) / USA (94) | Retrospective analysis of 2 cohorts / 522 | Various | No relation between surgery/ anaesthesia and decline in executive function but association with memory decline was found | Patients exposed to surgery were reported to have a more rapid deterioration on cognitive function | Exposure to surgery/ general anaesthesia, presence of APOE4 allele were found to have a synergistic effect in POCD incidence | NA |
| Inouye (2016) / USA (95) | Cohort / 560 | > 70 years  Various | While patients with POD showed a significant 1 month decline in cognition, when measured at 2 months, no significant difference was found | Cognitive function postoperative was measured until 36 months showing a gradual decline in cognition postop | Patients with POD developed a greater cognitive impairment in 1 month and 36 months postoperative | NA |
| Berger (2014) / USA (96) | Narrative review | NA | Study discusses the association between perioperative care, POD, POCD and Alzheimer’s disease | NA | Study suggest an important role for Alzheimer’s disease in POCD development | NA |
| Lundström (2003) / Sweden (97) | Cohort / 100 | Femoral neck fracture surgery | See antecedents column | NA | Patients with POD, DM and worse preoperative cognitive score developed higher rates of dementia 5 years postop | See antecedents |
| Pietzsch (2021) / Germany (98) | Model analysis | Various | POCD has a substantial burden on the long- term care insurance | NA | NA | Study estimated 288,375 cases of POCD yearly, with a financial yearly burden of 1.6 billion EUR for Germany |
| Boone (2020) / USA (99) | Cohort / 2,380,473 | Various | Postoperative neurocognitive disorder (PND) development increases costs 17,275 USD during the 1- year postop | NA | NA | Patients with PND had longer hospital stay and 1- year mortality and were less likely to be discharged home |
| Schmitt (2015) / USA (100) | Cohort / 566 | ≥ 70 years  Various | Assessment of POD was done using CAM and DSI and severity by CAM-S | | | |
| Kimchi (2017) / USA (101) | Systematic review | No surgery | Study noted the variability in diagnosing dementia and MCI in other studies and provides recommendation for expert panel constitution, providing data to said panel, decision process and validity diagnosis | | | |
| Whitlock (2011) / USA (102) | Narrative review | NA | Reviews POD’s epidemiology, diagnosis, prevention and treatment | Study discusses the use of CAM, and CAM-ICU for measuring POD | Study discusses the relation between POD and POCD | (surrogate term: *POD*). |
| Schubert (2018) / Switzerland (103) | Cohort / 10,906 | Some had surgery | Delirium incidence was found to be 24.8 %  Defines delirium as an acute mental change which affects consciousness, attention, cognition and psychomotor behavior and emotions (surrogate term: *POD*). | | | |
| Ho (2021) / Australia (104) | Systematic review and meta- analysis / 3,533 | Various | CAM and CAM-ICU was found to be the most prevalent tools used to assess POD. CAM was used in more than half of the studies and was also suggested to be the most efficient tool while being less time-consuming. Study also found the total incidence of POD to be 24% (surrogate term: *POD*). | | | |
| Funder (2010) / Denmark (105) | Narrative review | NA | Study discusses the methodological problems in neuropsychological testing (e.g. floor-ceiling effect, learning effect, loss to follow- up, drug effect, reliability of preoperative test and timing of preoperative testing | POD is an acute confusional state that is reversible and may present with altered level of consciousness.  Dementia may present also with behavioral and personality changes and should at least last 6 months | NA | NA |
| Gale (2018) / USA (106) | Narrative review | NA | Describes dementia as a syndrome which interferes with a person’s functioning because of a decline in cognitive function, which can be in essence reversible or irreversible (surrogate term: *Major Neurocognitive Disorder (Dementia)*). | | | |
| Arvanitakis (2019) / USA (107) | Narrative review | NA | Study finds that evaluating dementia requires medical history taking along neurologic examination and history should consist of nature, magnitude and course of cognitive decline (surrogate term: *Major Neurocognitive Disorder (Dementia)*). | | | |
| Smits (2015) / Netherlands (108) | Cohort / 270 | No surgery | Baseline cognitive function was found to be impaired in all subtypes of dementia inspected (Alzheimer’s disease, vascular dementia, lewy body dementia, frontotemporal dementia) in all cognitive domains but visuospatial functioning was only affected in lewy body dementia and Alzheimer’s disease. In follow-up various results were obtained in different subtypes of dementia regarding the cognitive domain which decline happened (surrogate term: *Major Neurocognitive Disorder (Dementia)*). | | | |
| Petersen (2004) / USA (109) | Narrative review | NA | Study proposes a procedure for diagnosing MCI consisting of these steps: first determining the patient is neither demented nor of normal cognitive functioning, secondly through history taking cognitive decline could be found, then checking whether daily functioning is intact and lastly after considering MCI, the search for its subtypes (amnestic vs. non- amnestic) should be probed (surrogate term: *Mild Neurocognitive Disorder*). | | | |
| Petersen (2018) / USA (110) | Guideline | NA | MCI prevalence was found to be 6.7% for ages 60 – 64 years and prevalence increased by age to 25.2% for ages 80 - 84 years. MCI was more common in people with higher age and lower education. Although MCI can be reversible, people with MCI have a higher chance of progressing to dementia (surrogate term: *Mild Neurocognitive Disorder*). | | | |
| Domínguez (2019) / Mexico (111) | Concept analysis | NA | Study defines MCI as a possible reversible cognitive state somewhere between what is normal for age and early stages of dementia, where patient is capable of instrumental daily living activities while also impairment in cognitive domains is evident (surrogate term: *Mild Neurocognitive Disorder*). | | | |
| Jongsiriyanyong (2018) / Thailand (112) | Narrative review | NA | Study argues the outcome of MCI (reversing to normal, stabilizing or progressing to dementia) to be a result of the underlying problem, thus recommends investigations be done to discover the pathology leading to MCI in order to revers or procrastinate cognitive decline (surrogate term: *Mild Neurocognitive Disorder*). | | | |
| Tangalos (2018) / USA (113) | Narrative review | NA | Study argues that although diagnosing MCI requires a thorough examination consisting of neuropsychiatric testing and imaging, primarily to diagnose and secondly to search for the pathology, ultimately it should be understood that diagnosing MCI is a clinical matter, especially noteworthy in patients with a previously higher intellectual function that is currently complaining with a decrease in cognitive ability but cognitive testing provides normal for age results (surrogate term: *Mild Neurocognitive Disorder*). | | | |
| Harerimana (2020) / Canada (114) | Concept analysis | NA | Defines cognitive vitality the ability for an individual to adapt to cognitive changes while also being able to sustain life satisfaction, independent living and optimal cognitive functioning. This cognitive vitality acts as a protective compensating factor to cognitive decline during aging (surrogate term: *Cognitive Vitality*). | | | |
| McDonough (2015) / USA (115) | Cohort / 39 | No surgery | Study found that engaging in challenging tasks improves cognitive domains such as attention and semantic processing by increasing the neural efficiency in temporal and parietal cortex (surrogate term: *Cognitive Vitality*). | | | |
| Gow (2005) / Scotland (116) | Cohort / 1921 | NA | Study found that happiness in non-demented elderly is not related to patients’ cognitive ability at age 11 and 79 (surrogate term: *Cognitive Vitality*). | | | |
